# Ginsenoside Rc attenuates myocardial ischaemic injury through antioxidative and anti-inflammatory effects

**DOI:** 10.1080/13880209.2022.2072518

**Published:** 2022-05-28

**Authors:** Lei Shi, Wenwen Fu, Huali Xu, Shihui Li, Xinyu Yang, Wei Yang, Dayun Sui, Quanwei Wang

**Affiliations:** a Department of Cardiovascular Medicine, First Hospital, Jilin University, Jilin, PR China; b Department of Pharmacology, School of Pharmacy, Jilin University, Jilin, PR China

**Keywords:** *Panax ginseng*, Nrf2 inhibitor, inflammatory cells infiltration, oxidative stress

## Abstract

**Context:**

*Panax ginseng* C. A. Meyer (Araliaceae) is a famous Asian medicine. Ginsenoside Rc is a component isolated from *Panax ginseng*.

**Objective:**

This study evaluates the effect of ginsenoside Rc on myocardial ischaemic injury.

**Materials and methods:**

Male Swiss mice were subcutaneously injected with 50 mg/kg isoproterenol once a day for three days. Ginsenoside Rc (10, 20, or 40 mg/kg) was intragastrically administered 1 h after isoproterenol injection. The mice in the control group were subcutaneously injected with normal saline and intragastrically given 0.5% CMC-Na. CK-MB and troponin T were assayed. Histopathological examination of myocardium was conducted. The expression of Nrf2, GCLC, GCLM and HO-1 in heart tissues was evaluated by Western blot.

**Results:**

In myocardial ischaemic mice, ginsenoside Rc reduced the levels of CK-MB (197.1 ± 15.7, 189.9 ± 19.0, 184.0 ± 14.4 vs. 221.6 ± 27.9) and troponin T (10.3 ± 1.7, 9.5 ± 1.3, 8.7 ± 1.7 vs. 13.4 ± 2.4). Ginsenoside Rc attenuated the necrosis and inflammatory cells infiltration in myocardium. Furthermore, ginsenoside Rc not only decreased the contents of MDA, TNF-α but also increased GSH level in the heart tissues. The expression of Nrf2, GCLC, GCLM and HO-1 was significantly increased in the animals treated with ginsenoside Rc. ML385, an Nrf2 inhibitor, blocked partially the ginsenoside Rc-mediated cardioprotective effect. Ginsenoside Rc attenuated myocardial ischaemic injury in mice, which may be, in part, through its antioxidative and anti-inflammatory effects.

**Conclusions:**

This study indicated that ginsenoside Rc might be a novel candidate for treatment of myocardial ischaemia.

## Introduction

Myocardial ischaemia is the leading cause of death and disability worldwide. Myocardial ischaemia is caused by an imbalance between myocardial oxygen demand and supply. It results in the myocardial pathological changes ranging from injury to necrosis. Myocardial ischaemia affects the diastolic properties of heart by slowing ventricular relaxation and increasing ventricular stiffness. Thus, it frequently leads to systolic and diastolic dysfunction (Ihara et al. 1994). Myocardial ischaemia also may precipitate heart failure exacerbation. It should be noted that myocardial ischaemia can be either a cause or a consequence of heart failure. Previous study demonstrated that about 70% of heart failure patients have ischaemic heart disease (Pagliaro et al. 2020). Myocardial ischaemia is the most common cause of lethal arrhythmias including ventricular tachycardia and fibrillation. A significant proportion of mortality in myocardial ischaemia patients is due to sudden cardiac death caused by ventricular arrhythmias (Agewall 2017).

Oxidative stress plays an important role in the cardiomyocyte injury induced by myocardial ischaemia. Reactive oxygen species (ROS) production will increase after myocardial ischaemia (Younis and Mohamed 2021). The decrease of endogenous ROS scavenger is attributed to the augment of ROS. ROS causes the injury of myocardial cells either through leading to lipid peroxidation, protein carbonylation and DNA oxidation or acting as a signal molecule in the cell death signalling pathway (Shabab et al. 2021). The process of myocardial ischaemia also leads to the activation of leukocyte and recruitment of neutrophils to the myocardium, following the production of cytokines (Ruparelia et al. 2017). Cytokines, such as IL-1β and TNF-α, are closely related to the pathophysiology of the injury of myocardial ischaemia. For example, TNF-α can induce releases of chemokines and adhesion molecules in the ischaemic heart tissues, promoting further leukocyte infiltration (Tang et al. 2018). IL-1β is also upregulated after myocardial ischaemia and leads to the injurious processes mentioned above (Gan et al. 2018).

*Panax ginseng* C. A. Meyer (Araliaceae) (ginseng) is a well-known traditional Chinese medicine. Recent studies demonstrated that there are many active components in ginseng showing cardioprotective effects. Ginsenosides extracted from ginseng are considered as bioactive constituents which are promising agents for treatment of cardiovascular diseases and stroke (Wang et al. 2021; Xie et al. 2021). Ginsenoside Rc belongs to the type of protopanaxadiol ginsenosides. Previous study showed that ginsenoside Rc upregulated catalase by regulating FoxO1 phosphorylation or FoxO1 acetylation. Thus, it suppressed ROS production and ameliorated the oxidative stress-induced injury in human embryo kidney 293 T cells (Kim et al. 2014). In lipopolysaccharide-challenged macrophages, ginsenoside Rc showed an anti-inflammatory property by regulating TANK-binding kinase 1 signalling pathway (Yu et al. 2017). It was shown that ginsenoside Rc could improve energy metabolism of cardiomyocytes, neurons and attenuated the injuries of myocardial or cerebral ischaemia (Huang et al. 2021). Ginsenoside Rc attenuated the acute cold exposure-induced myocardial injury in rats. The mechanisms of action were partially associated with the regulation of mRNA levels of TNF-α, IL-1β and IL-6 in myocardium (Xue et al. 2021). However, it is still not clear whether ginsenoside Rc can attenuate isoproterenol-induced myocardial ischaemic injury. This study aims to evaluate the effect of ginsenoside Rc on myocardial ischaemic injury in mice.

## Materials and methods

### Ginsenoside Rc and chemical reagents

Ginsenoside Rc was purchased from Yuanye Bio-technology Co., Ltd (Lot number: M27GB141849, purity > 98%). Isoproterenol and ML385 (Nrf2 inhibitor) were bought from Sigma Co. (St. Louis, MO, USA). Carboxymethyl cellulose sodium salt (CMC-Na) was from Shanghai Jinshan Chemical Co., Ltd (Shanghai, China). Creatine kinase (CK-MB, Lot number: 20210518), malondialdehyde (MDA, Lot number: 20210621) and glutathione (GSH, Lot number: 20210624) biochemical assay kits were obtained from Nanjing Jiancheng Bioengineering Institute (Nanjing, China). TNF-α (Lot number: M21015903) and IL-1β (Lot number: V21013165) ELISA kits were from Wuhan Huamei Biotech Co., Ltd (Wuhan, China). Troponin T ELISA kit (Lot number: 20210618) was purchased from Solarbio Science & Technology Co., Ltd (Beijing, China). Nuclear and cytoplasmic protein extraction kit, BCA protein kit and ECL detection reagents were from Beyotime Institute of Biotechnology (Shanghai, China). Other reagents were of commercially analytical grade.

### Animals

Male Swiss mice weighing 20–24 g were from Jinan Pengyue laboratory animal breeding Co., Ltd (Jinan, China). The mice were housed in cages under a control condition (12-h light/dark cycle, 22 ± 1 °C). The animals were allowed free access to food and water. The experimental procedures were conducted in accordance with the principles outlined in the NIH Guide for the Care and Use of Laboratory Animals and were approved by the Institutional Animal Care and Use Committee of the Jilin University (Authorization Number: 20210609).

### Experimental protocols

Experiment 1: To evaluate whether gensenoside Rc alone can cause myocardium injury in normal mice. Mice were randomly divided into four groups: control, gensenoside Rc at 10, 20, or 40 mg/kg groups. The animals were intragastrically administered with gensenoside Rc, whereas the mice in control group were given 0.5% CMC-Na. Once a day for three days. Then the levels of CK-MB and troponin T in serum were assayed. And the histopathological examination of myocardium was performed.

Experiment 2: To evaluate whether gensenoside Rc can attenuate myocardium injury in myocardial ischaemic mice. Mice were randomly divided into five groups: control, isoproterenol, gensenoside Rc at 10, 20, or 40 mg/kg groups. The mice were subcutaneously injected with isoproterenol at dose of 50 mg/kg, once a day for three days. Ginsenoside Rc was intragastrically administered at 1 h after isoproterenol injection. At 24 h after the last isoproterenol injection, CK-MB and troponin T were assayed. Histopathological examination of myocardium was conducted. MDA, GSH, IL-1β, TNF-α and the expression of Nrf2, GCLC, GCLM and HO-1 in heart tissues were evaluated.

Experiment 3：To evaluate whether Nrf2 inhibitor can abate the gensenoside Rc-mediated cardioprotective effect. Mice were randomly divided into four groups: control, isoproterenol, gensenoside Rc at 20 mg/kg and gensenoside Rc (20 mg/kg) plus ML385 groups. Mice were subcutaneously injected with isoproterenol at dose of 50 mg/kg, once a day for three days. Ginsenoside Rc was intragastrically administered at 1 h after isoproterenol injection. At the same time, the animals in gensenoside Rc plus ML385 group were also intraperitoneally injected with ML385 at dose if 30 mg/kg. At 24 h after the last isoproterenol injection, the parameters mentioned in Experiment 2 were evaluated.

### Assay of CK-MB and troponin T

The mice were anaesthetized with isoflurane. The blood was collected. The samples of blood were left at 4 °C for 1 h and then centrifuged (3500 *g*) at 4 °C for 15 min. The serum was harvested and stored at −80 °C for biochemical assay. The CK-MB and troponin T were assayed according to the manufacturer's instructions.

### MDA, GSH, IL-1β and TNF-α measurements

At the end of the experiment, the hearts of eight mice in each group were harvested. The hearts were washed three times with phosphate buffer and then homogenized in 4:1 ratio of cold phosphate buffer to heart tissue mass. The homogenate was centrifuged at 4000 *g* for 10 min at 4 °C. The total protein of the supernatants was assayed the BCA protein kit. The supernatants were stored at −80 °C and used to measure MDA, GSH, IL-1β and TNF-α in heart tissues.

### Histopathological examination

The hearts of three mice in each group were fixed with 10% formalin for 24 h. The specimens were embedded with paraffin and then were cut into 5 μm thick sections. The heart sections were stained using the method of hematoxylin-eosin staining. The histopathological changes were observed by an experimenter who was blinded to the groups under an Olympus microscope (IX-70, Olympus Corp., Japan).

### Western blot

Three hearts in each group were collected. Protein from the nucleus and cytoplasm of the heart tissues were prepared with the nuclear and cytoplasmic protein extraction kit. The protein content was assayed using the BCA protein kit. The sample (50 μg) was loaded onto 8% sodium dodecyl sulfate-polyacrylamide gel electrophoresis. Proteins were transferred to polyvinylidene fluoride membranes for 1 h at 120 V. The membranes were incubated overnight at 4 °C with primary antibodies: rabbit anti-Nrf2 (1:1000, Abcam, MA, USA), rabbit anti-glutamate cysteine ligase catalytic subunit (GCLC) (1:3000, Abcam, MA, USA), rabbit anti-glutamate cysteine ligase modifier subunit (GCLM) (1:3000, Abcam, MA, USA), or rabbit anti-HO-1 (1:2500, Abcam, MA, USA). The membranes were processed with the corresponding horseradish peroxidase-labeled secondary antibody (1:3000, Beyotime Institute of Biotechnology, Shanghai, China). Bands were visualized with the ECL detection reagents. The relative density of protein was analysed using ImageJ software (Media Cybernetics, Silver Spring, MD, USA). The ratio of the density of the detected protein to that of Lamin B1 or β-actin was used for statistical analysis.

### Statistical analysis

All data were expressed as mean ± SD and processed with SPSS 22.0. The data were analysed with one-way ANOVA followed by Tukey’s *post hoc* test. Statistical significance was defined as *p* < 0.05.

## Results

### Effect of ginsenoside Rc on CK-MB, troponin T and histopathological structure in normal mice

**Figure 1.**
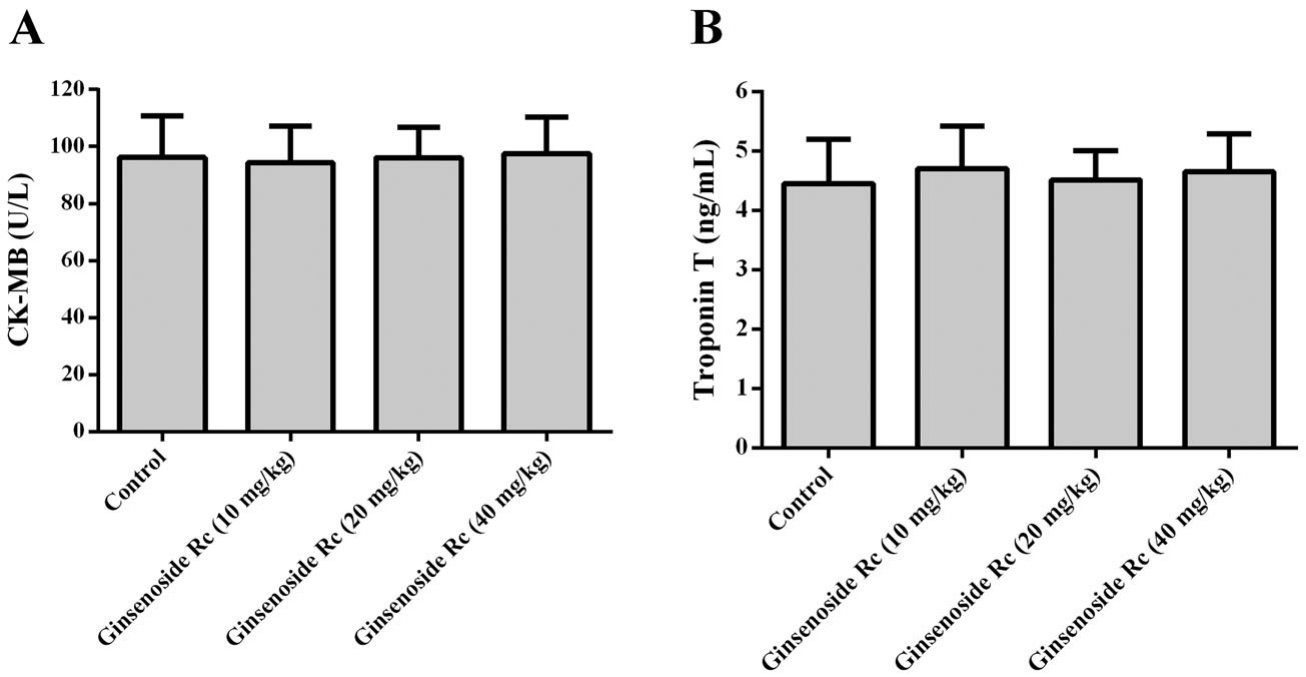
Effect of ginsenoside Rc on CK-MB and troponin T in normal mice. (A) Ginsenoside Rc had no effect on CK-MB in normal mice. (B) Ginsenoside Rc had no effect on troponin T in normal mice. Data are presented as the mean ± SD (*n* = 8).

**Figure 2.**
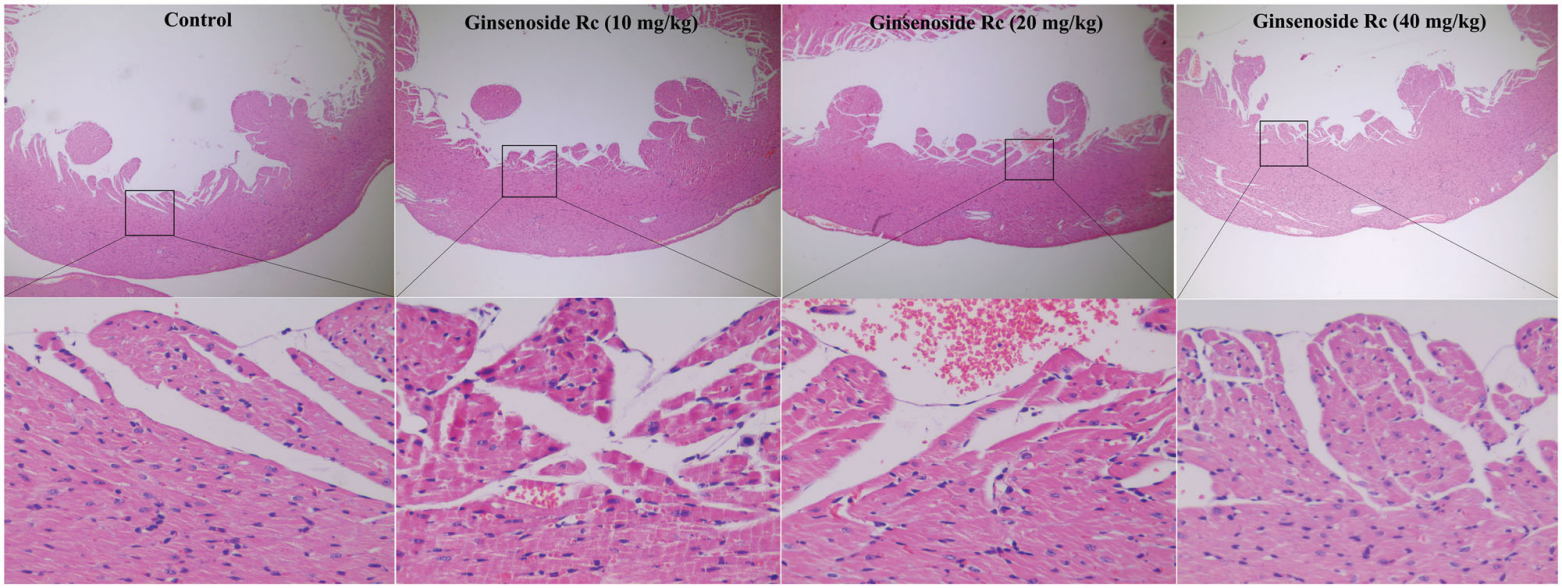
Effect of ginsenoside Rc on histopathological structure in normal mice. The myocardia of the top (×100) and the bottom (×400) are at different magnifications (*n* = 3).

Compared with the control group, the CK-MB and troponin T of ginsenoside Rc groups did not show significant changes (Figure 1, *p* > 0.05). Further histopathological examination demonstrated that myocardial structure including endocardium, myocardium and epicardium in ginsenoside Rc groups is normal (Figure 2). These findings indicated that ginsenoside Rc alone did not cause myocardium injury in normal mice.

### Effect of ginsenoside Rc on CK-MB and troponin T in myocardial ischaemic mice

**Figure 3.**
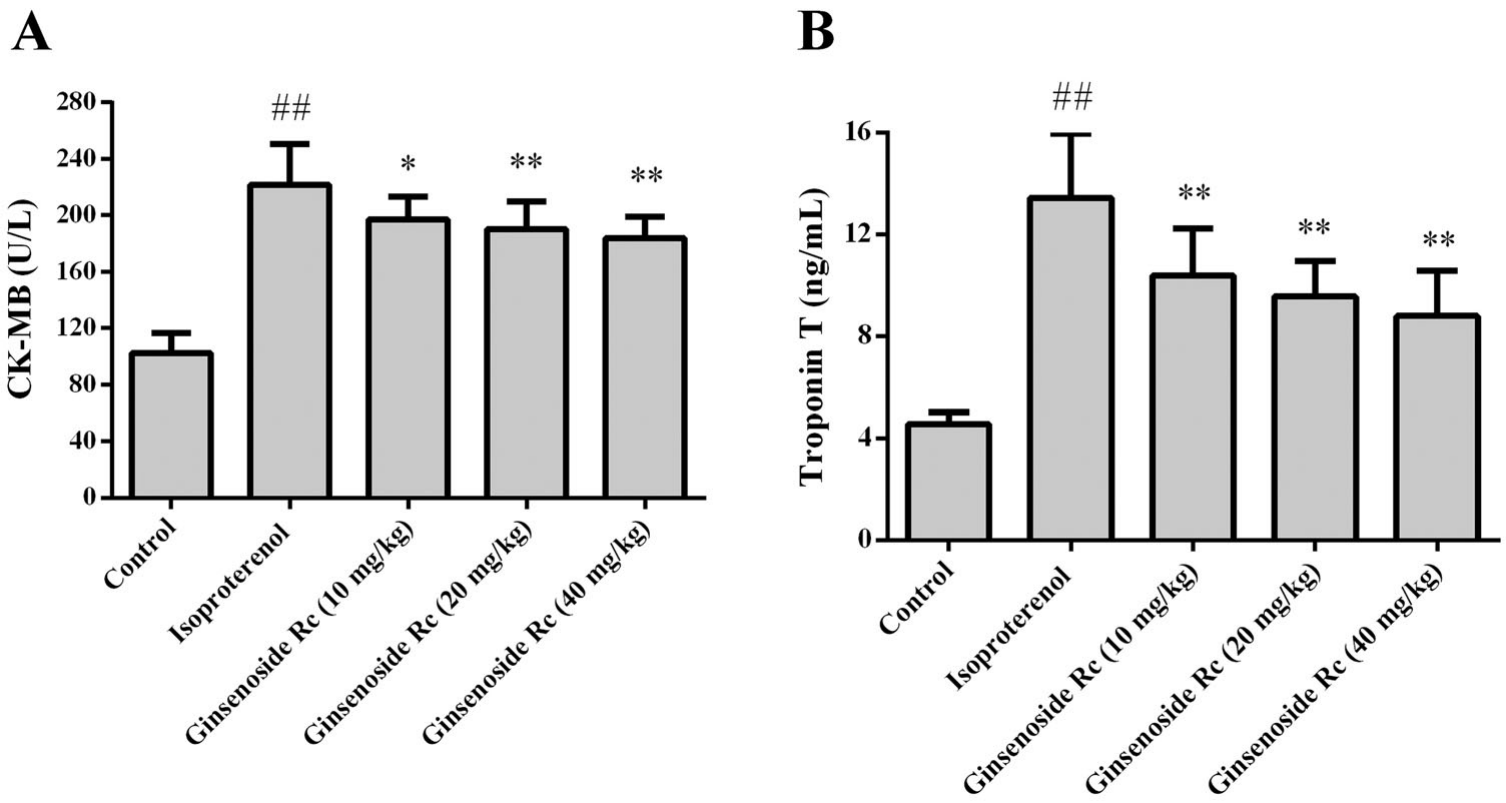
Effect of ginsenoside Rc on CK-MB and troponin T in myocardial ischaemic mice. (A) CK-MB. (B) Troponin T. ^##^*p* < 0.01 compared with the control group; *p < 0.05, **p < 0.01 compared with the isoproterenol group. Data are presented as the mean ± SD (*n* = 14).

The CK-MB and troponin T of the animals in the isoproterenol group were significantly augmented as compared with that of the control group (*p* < 0.05 or *p* < 0.01). Compared with the isoproterenol group, the CK-MB and troponin T of the mice in the ginsenoside Rc at dose of 10, 20, or 40 mg/kg groups were reduced (*p* < 0.05 or *p* < 0.01) (Figure 3).

### Effect of ginsenoside Rc on histopathological structure in myocardial ischaemic mice

**Figure 4.**
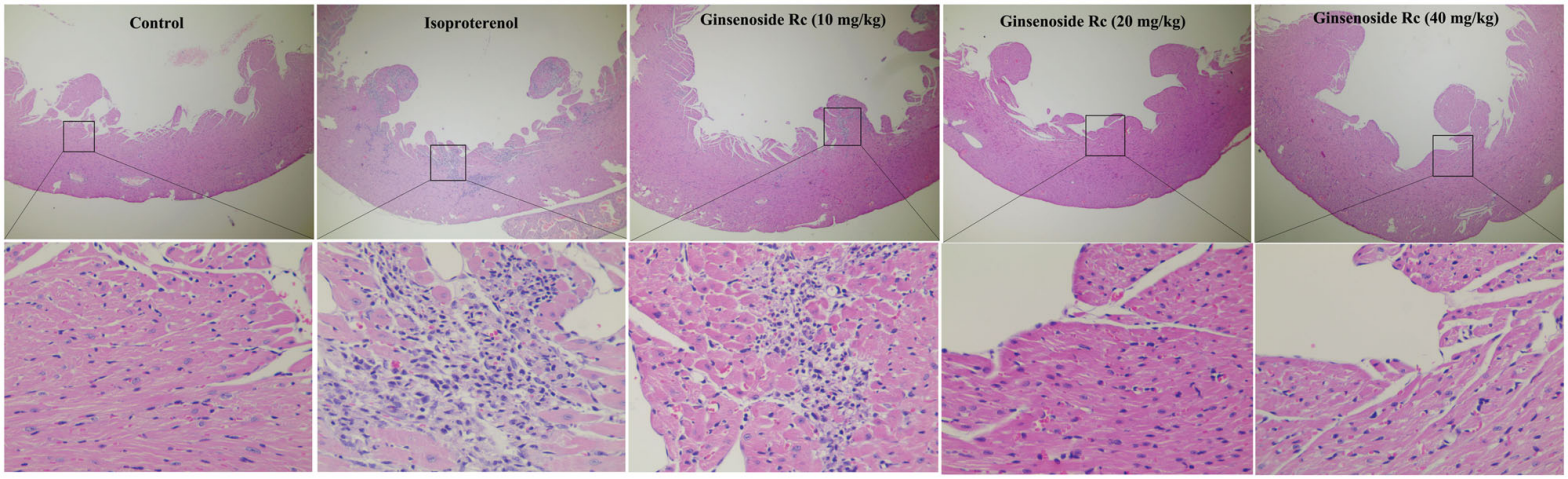
Effect of ginsenoside Rc on histopathological structure in myocardial ischaemic mice. The myocardia of the top (×100) and the bottom (×400) are at different magnifications (*n* = 3).

In the control group, the histological structure of myocardium is normal. The myocardial cells showed a clear integrity and there were no inflammatory cells infiltration observed. The myocardium of mice in the isoproterenol group displayed focal necrosis and myofibrillar fracture with inflammatory cell infiltration. However, the histopathological changes in myocardium of the ginsenoside Rc groups were significantly alleviated. The inflammatory cell infiltration and the degree of focal necrosis and myofibrillar fracture were attenuated (Figure 4). These results demonstrated that ginsenoside Rc has a property of attenuating myocardial ischaemic injury.

### Effect of ginsenoside Rc on MDA, GSH, IL-1β and TNF-α in myocardial ischaemic mice

**Figure 5.**
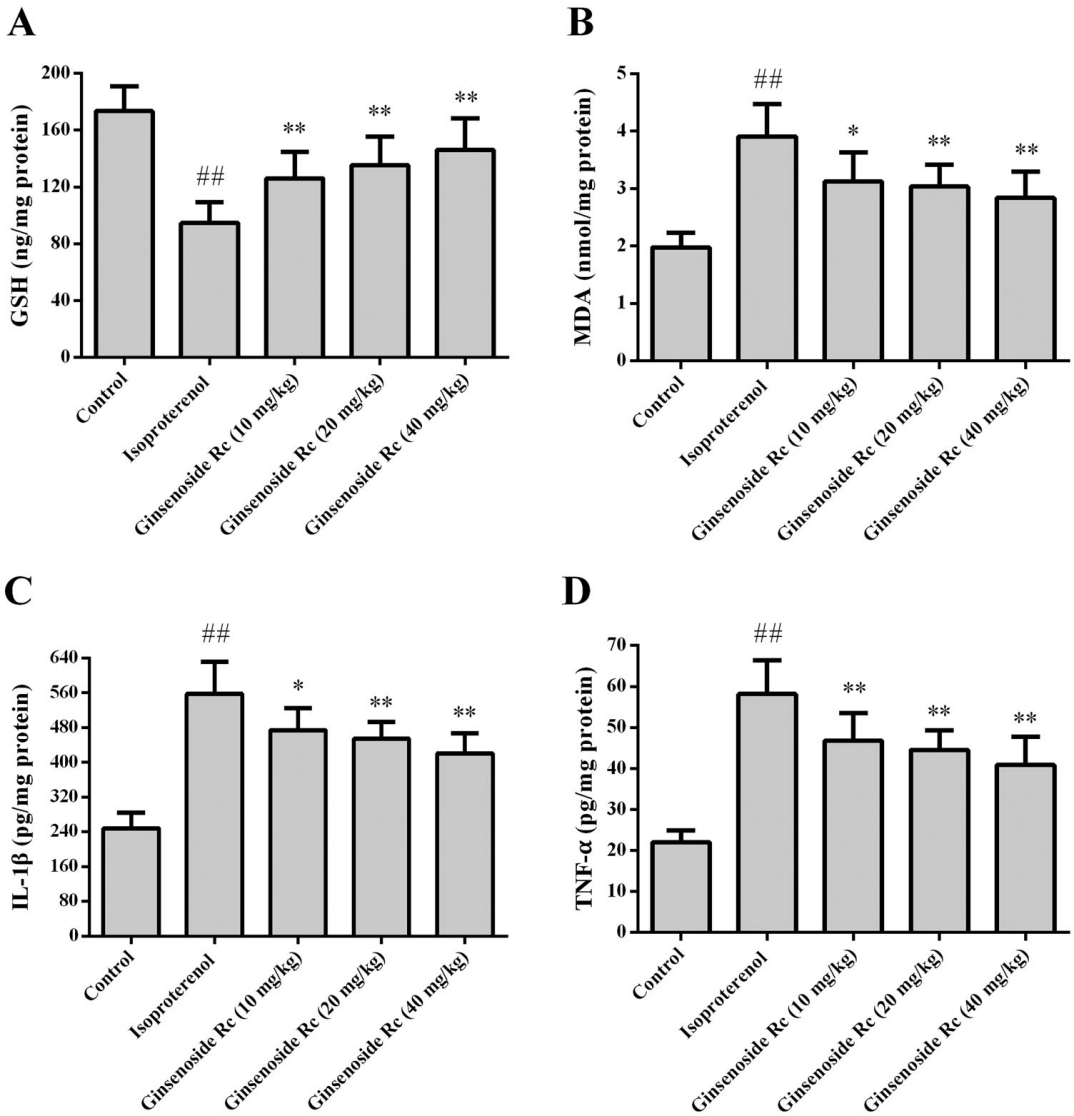
Effect of ginsenoside Rc on GSH, MDA, IL-1β and TNF-α in myocardial ischaemic mice. (A) GSH. (B) MDA. (C) IL-1β. (D) TNF-α. ^##^*p* < 0.01 compared with the control group; *p < 0.05, **p < 0.01 compared with the isoproterenol group. Data are presented as the mean ± SD (*n* = 8).

Figure 5 shows the effect of ginsenoside Rc on MDA, GSH, IL-1β and TNF-α in myocardial ischaemic mice. Compared with the control group, the levels of MDA, GSH, IL-1β and TNF-α in the myocardium of the isoproterenol group were significantly increased (*p* < 0.01). However, treatment with ginsenoside Rc markedly decreased the myocardial ischaemia-induced elevations of MDA, IL-1β and TNF-α (*p* < 0.05, *p* < 0.01). Compared with the control group, the level of GSH was decreased following isoproterenol injection (*p* < 0.01). Ginsenoside Rc treatment attenuated the reduction in GSH content (*p* < 0.01).

### Effect of ginsenoside Rc on expression of Nrf2, GCLC, GCLM, HO-1 in myocardial ischaemic mice

**Figure 6.**
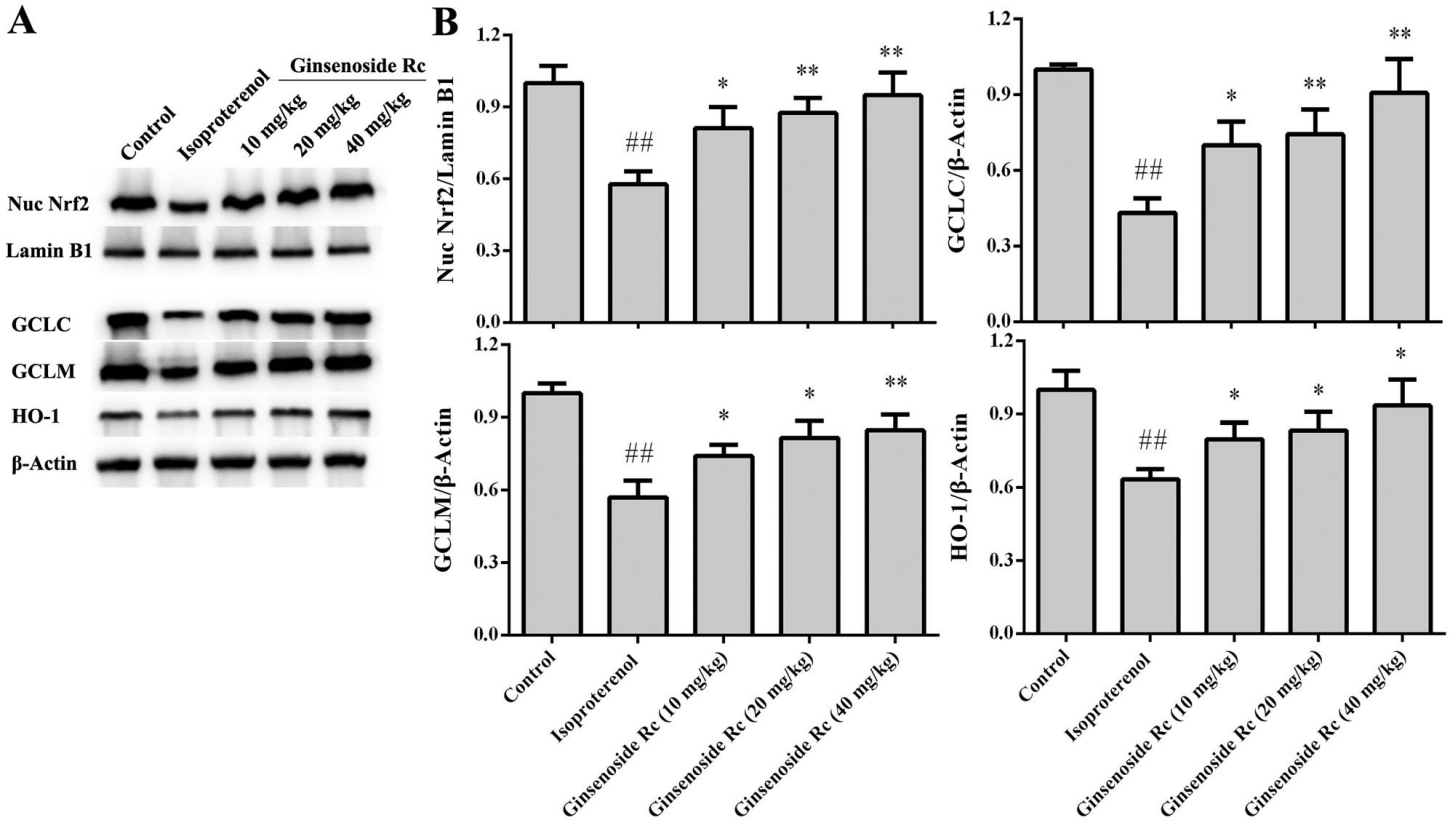
Effect of ginsenoside Rc on expression of Nrf2, GCLC, GCLM and HO-1 in myocardial ischaemic mice. (A) Representative photographs of the expression of Nrf2, GCLC, GCLM, HO-1. (B) Bar graphs of quantitative analysis of Nrf2, GCLC, GCLM, HO-1, respectively. ^##^*p* < 0.01 compared with the control group; *p < 0.05, **p < 0.01 compared with the isoproterenol group. Data are presented as the mean ± SD (*n* = 3).

In the isoproterenol group, the expression of Nrf2, GCLC, GCLM, HO-1 was decreased markedly when compared with the control group (*p* < 0.01). However, ginsenoside Rc treatment increased significantly the expression of Nrf2, GCLC, GCLM, HO-1 in myocardium (*p* < 0.05, *p* < 0.01) (Figure 6).

### Effect of Nrf2 inhibitor on cardioprotective effect of ginsenoside Rc

**Figure 7.**
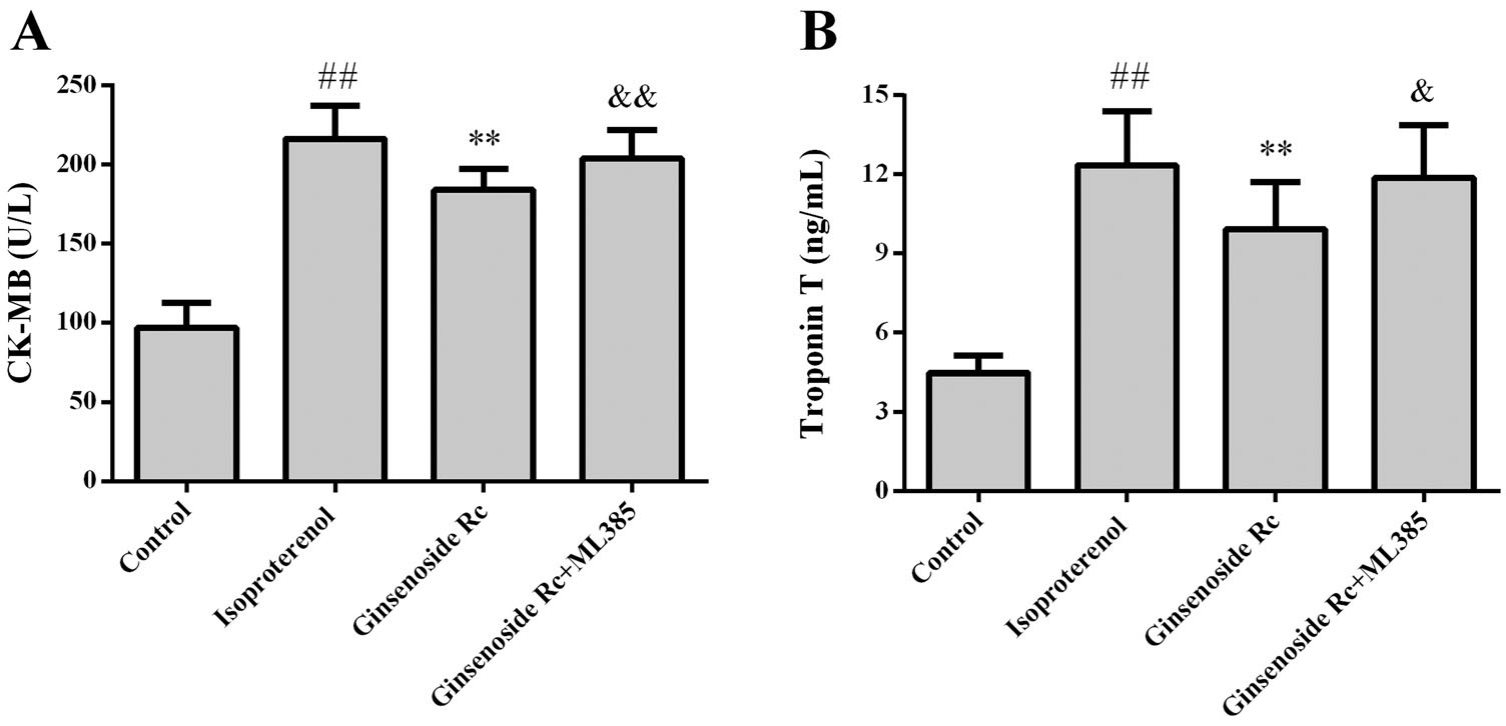
Effect of Nrf2 inhibitor on ginsenoside Rc-mediated decreases of CK-MB and troponin T in myocardial ischaemic mice. (A) CK-MB. (B) troponin T. ^##^*p* < 0.01 compared with the control group; **p < 0.01 compared with the isoproterenol group; ^&^*p* < 0.05, ^&&^*p* < 0.01 compared with the ginsenoside Rc group. Data are presented as the mean ± SD (*n* = 14).

**Figure 8.**
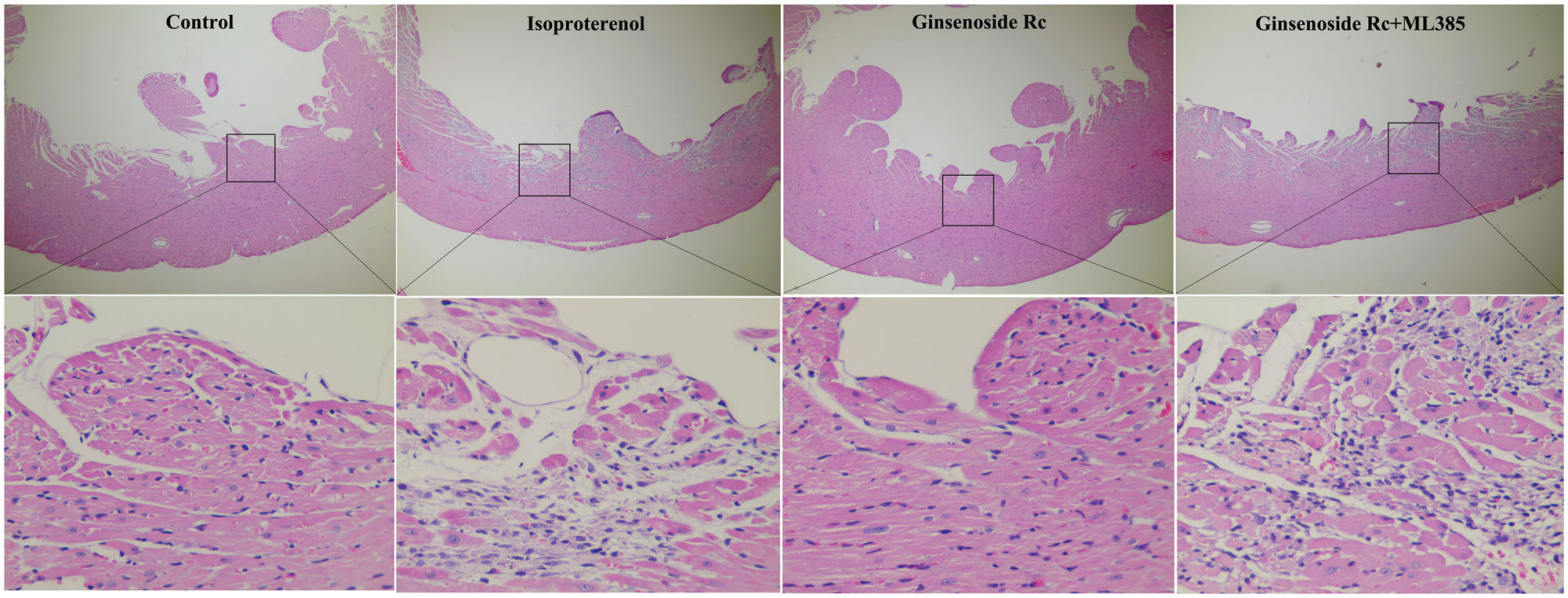
Effect of Nrf2 inhibitor on ginsenoside Rc-mediated improvement of histopathological structure changes in myocardial ischaemic mice. The myocardia of the top (×100) and the bottom (×400) are at different magnifications (*n* = 3).

The effect of ML385 on the ginsenoside Rc-mediated cardioprotective effect was evaluated. Ginsenoside Rc decreased the CK-MB, troponin T (*p* < 0.01) and attenuated the histopathological changes in myocardium induced by ischaemia. However, co-administration with ML385 reversed the ginsenoside Rc-mediated decreases of CK-MB, troponin T (*p* < 0.05, *p* < 0.01) and improvement of histopathological changes in myocardium (Figures 7 and 8).

### Effect of Nrf2 inhibitor on antioxidative effect of ginsenoside Rc

**Figure 9.**
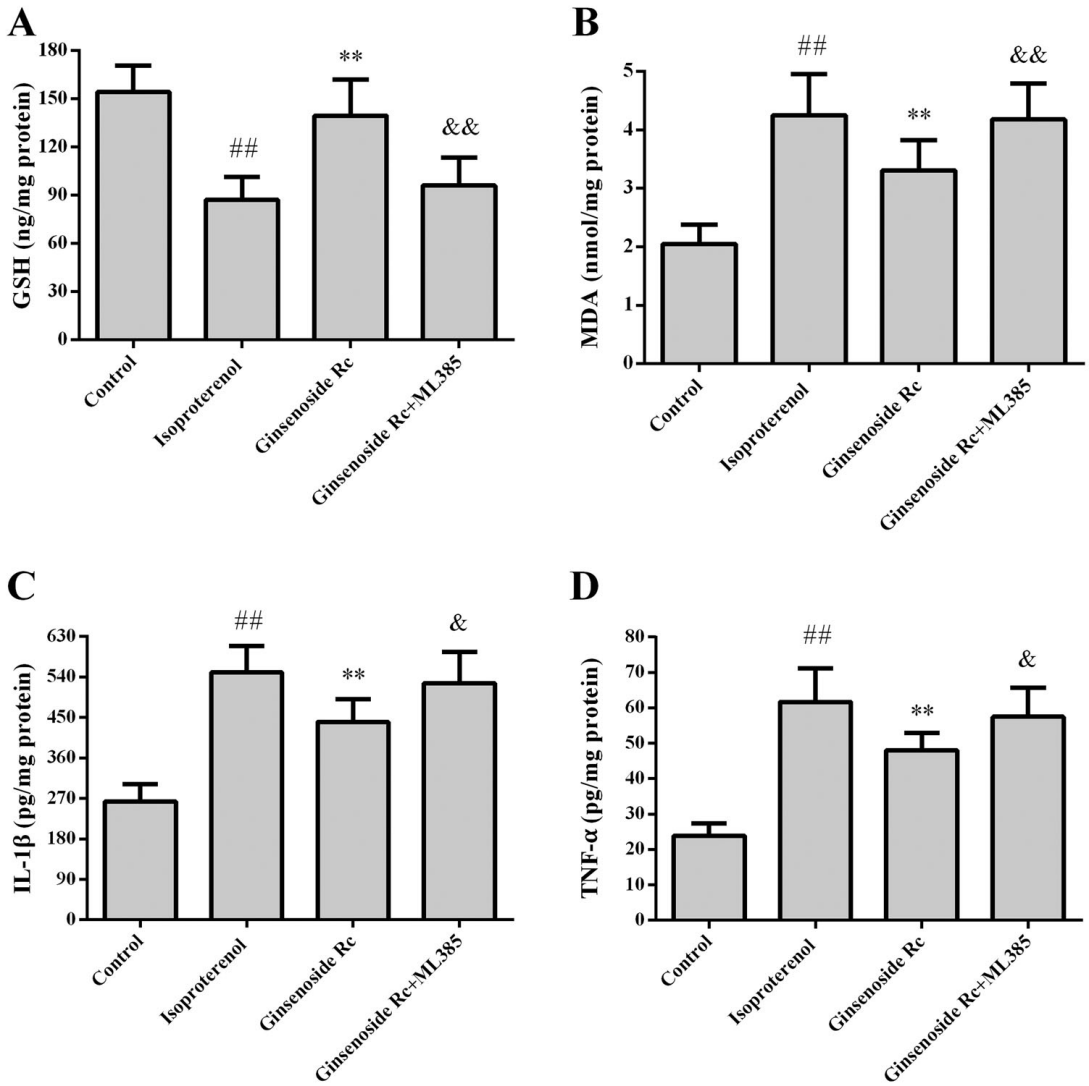
Effect of Nrf2 inhibitor on ginsenoside Rc-mediated improvement of changes of MDA, GSH, IL-1β and TNF-α in myocardial ischaemic mice. (A) GSH. (B)MDA. (C) IL-1β. (D) TNF-α. ^##^*p* < 0.01 compared with the control group; **p < 0.01 compared with the isoproterenol group; ^&^*p* < 0.05, ^&&^*p* < 0.01 compared with the ginsenoside Rc group. Data are presented as the mean ± SD (*n* = 8).

**Figure 10.**
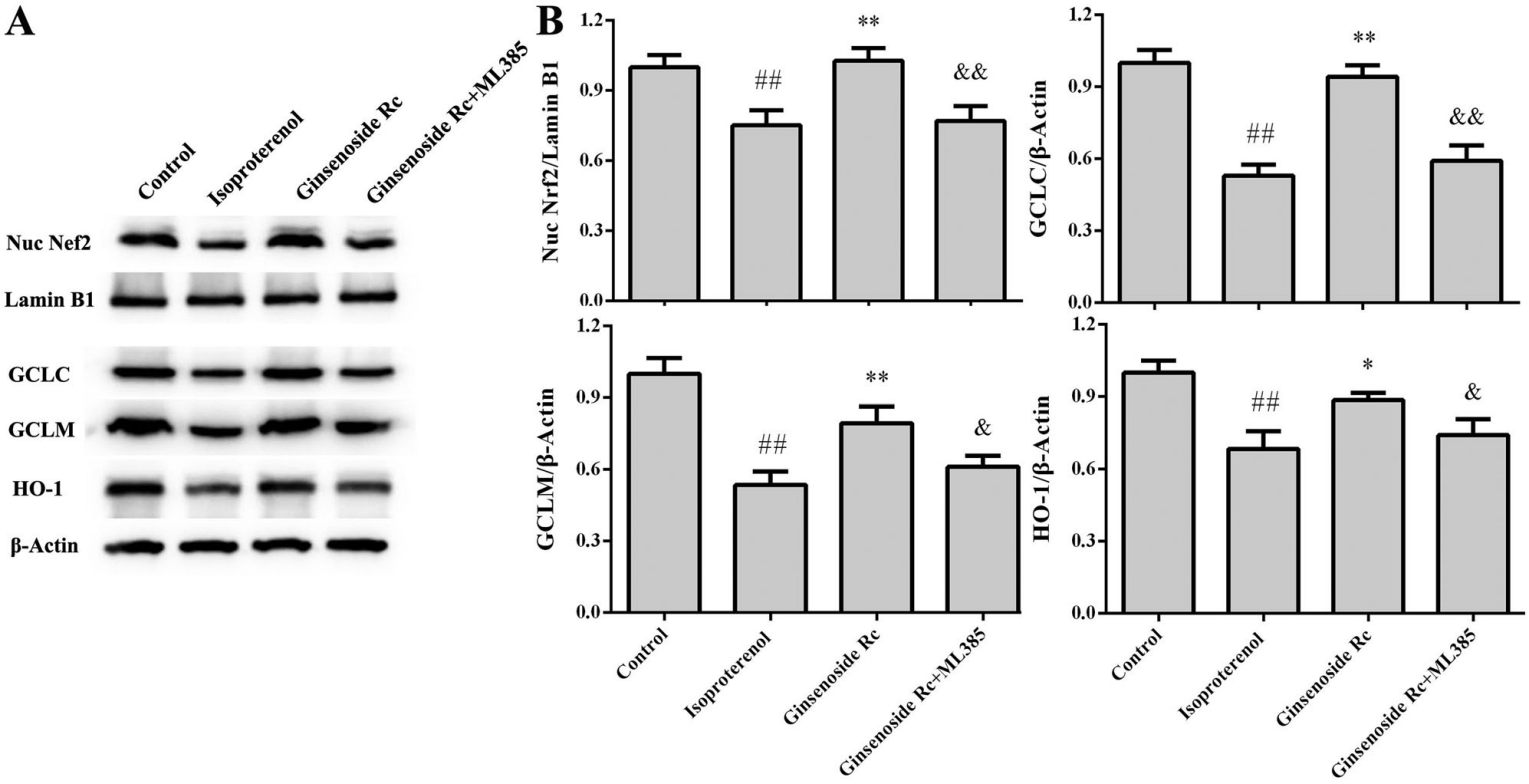
Effect of Nrf2 inhibitor on ginsenoside Rc-mediated regulation of Nrf2, GCLC, GCLM, HO-1 in myocardial ischaemic mice. (A) Representative photographs of the expression of Nrf2, GCLC, GCLM, HO-1. (B) Bar graphs of quantitative analysis of Nrf2, GCLC, GCLM, HO-1, respectively. ^##^*p* < 0.01 compared with the control group; *p < 0.05, **p < 0.01 compared with the isoproterenol group; ^&^*p* < 0.05, ^&&^*p* < 0.01 compared with the ginsenoside Rc group. Data are presented as the mean ± SD (*n* = 3).

To further confirm that the mechanism of action of ginsenoside Rc is associated with the Nrf2-regulated antioxidative effect, the mice were subjected to ML385. Ginsenoside Rc not only decreased the MDA (*p* < 0.01) but also increased GSH level and expression of Nrf2, GCLC, GCLM in myocardial ischaemic mice (*p* < 0.01). Compared with the ginsenoside Rc group, ML385 abated partially the ginsenoside Rc-mediated reduction of MDA (*p* < 0.01) and augments of GSH, Nrf2, GCLC, GCLM (*p* < 0.05, *p* < 0.01), (Figures 9(A, B) and 10)).

### Effect of Nrf2 inhibitor on anti-inflammatory effect of ginsenoside Rc

The effect of Nrf2 inhibitor on the anti-inflammatory property of ginsenoside Rc is shown in Figures 9(C, D) and Figure 10. In line with previous results, ginsenoside Rc reduced the myocardial ischaemia-induced elevations of IL-1β and TNF-α when compared with the isoproterenol group (*p* < 0.01). Ginsenoside Rc also augmented the expression of Nrf2 and HO-1 in myocardium (*p* < 0.05, *p* < 0.01). However, co-administration with ML385 reversed not only the ginsenoside Rc-mediated reductions of IL-1β and TNF-α (*p* < 0.05) but also the ginsenoside Rc-mediated elevation in the expression of Nrf2 and HO-1 (*p* < 0.05, *p* < 0.01).

## Discussion

Firstly, this study suggested that ginsenoside Rc did not show any adverse effect on the myocardium of the normal mice. Then, the isoproterenol-induced myocardial ischaemic model was used to evaluate the cardioprotective effects of ginsenoside Rc. The results showed that ginsenoside Rc could attenuate the ischaemia-induced myocardium injury. Further results demonstrated that ginsenoside Rc regulated the expression of Nrf2, GCLC, GCLM, HO-1 and therefore decreased MDA, IL-1β and TNF-α. Based on these findings, it is reasonable to conclude that the mechanisms of action of ginsenoside Rc are closely related to its antioxidative and anti-inflammatory effects.

Preclinical models are widely used to evaluate the cardioprotective potential of extracts of traditional Chinese medicine. The common animal models of myocardial ischaemia include surgery-induced model and chemical-induced model (Sorop et al. 2020). The mortality of surgery-induced model is higher than that of chemical-induced model. Isoproterenol exhibits agonist activity at β receptors. Isoproterenol leads to myocardial ischaemia in animals, which is similar to that of human. Isoproterenol causes inflammation, injury and necrosis in myocardium of mice (Huang et al. 2019). It demonstrated that isoproterenol can lead to the generation of free radicals, which cause progressive mitochondrial damage and myocardium injury. The inotropic and chronotropic effects of large doses of isoproterenol cause myocardial necrosis, inflammatory cell infiltration and myocardial fibrosis (Blas-Valdivia et al. 2022). Therefore, isoproterenol-induced myocardial ischaemic model is widely used to evaluate the cardioprotective effect of bioactive constituents of traditional Chinese medicine. This study showed that isoproterenol injection caused a significant elevation in CK-MB and troponin T. Administration of ginsenoside Rc abated the isoproterenol-induced increases of CK-MB and troponin T. In the histopathological examination, ginsenoside Rc also alleviated the isoproterenol-induced myocardium injury, such as myofibrillar fracture, neutrophil infiltration and necrosis. These results demonstrate that ginsenoside Rc has a cardioprotective property in myocardial ischaemia.

Nrf2 is a transcription factor that plays an important role in protecting cells in detrimental stresses (Chen 2021). If oxidative stress occurs, Nrf2 will translocate in the nucleus and then binds to antioxidant response element (ARE), regulating the expression of cytoprotective genes. Glutamate-cysteine ligase (GCL), composed of catalytic subunit GCLC and regulatory subunit GCLM, is a rate-limiting enzyme for GSH synthesis. GSH acts as a substrate for eliminating ROS and lipid peroxide. It is well-known that GCLC and GCLM contain ARE in their promoter regions and they are upregulated by Nrf2 activation (Lee et al. 2021).

Inflammation is accompanied with myocardium injury after ischaemia. The pathological inflammatory process leads to the generation of ROS that damage macromolecules in cardiomyocytes (Abdelzaher et al. 2021). Accumulating evidence demonstrate that the Nrf2/HO-1 signalling plays a pivotal role in regulating inflammation (Khalil et al. 2021). HO-1 is an inducible rate-limiting enzyme catalysing haem into CO. CO can inhibit NF-ĸB pathway and therefore attenuate the production of pro-inflammatory cytokines. Importantly, HO-1 directly not only inhibits proinflammatory cytokines but also activates anti-inflammatory cytokines, thus regulating the inflammatory process (Chen 2021). Nrf2 induces the HO-1 protein expression and HO-1 is one of the classic Nrf2-regulated genes. Studies demonstrated that activation of Nrf2/HO-1 signalling reduced TNF-α, IL-1β and IL-6 levels, and consequently inhibited the inflammatory reaction (Kim et al. 2021). In this study, MDA, IL-1β and TNF-α were increased, whereas GSH was significantly reduced in myocardial ischaemic mice. Ginsenoside Rc elevated the activation of Nrf2, which augmented the expression of GCLC and GCLM. GCL promoted GSH synthesis, which attenuated the oxidative stress injury of myocardium. The ginsenoside Rc-involved Nrf2 activation also upregulated the expression of HO-1. Consistent with the results mentioned above, the levels of IL-1β and TNF-α in myocardium were decreased when ginsenoside Rc increased the expression of Nrf2 and HO-1 in myocardium. These findings indicated that the cardioprotective property of ginsenoside Rc was associated with its antioxidative and anti-inflammatory effects.

ML385, an Nrf2 inhibitor, can block Nrf2 transcriptional activity (Tastan et al. 2021). To further corroborate our hypothesis, ML385 was co-administered with ginsenoside Rc treatment. In line with the findings mentioned in the preceding section, ML385 reversed the ginsenoside Rc-mediated cardioprotective effect and the ginsenoside Rc-mediated improvement of changes of MDA, GSH, IL-1β and TNF-α in myocardial ischaemic mice. Furthermore, ML385 also abolished the effect of ginsenoside Rc on the expression of Nrf2, GCLC, GCLM and HO-1. Therefore, it is reasonable to suggest that ginsenoside Rc attenuates myocardial ischaemic injury through its anti-inflammatory and antioxidative effects and Nrf2 plays a key role in the ginsenoside Rc-mediated antioxidative and anti-inflammatory effects.

NF-κB is a protein complex involving in the process of inflammation. It is supposed that Nrf2 and NF-κB signalling pathways may interact to regulate the cellular response to myocardial ischaemia (Feng et al. 2021). However, this study did not investigate the interplay of Nrf2 and NF-kB in the cardioprotective effect of ginsenoside Rc. This is the limitation of the present study.

## Conclusions

The present experiment demonstrated that ginsenoside Rc attenuated myocardial ischaemic injury in mice, which may be, in part, through its antioxidative and anti-inflammatory effects. This study indicated that ginsenoside Rc might be a novel candidate for the treatment of myocardial ischaemia.

## Geolocation information

Changchun is at 125.35 degrees East longitude and 43.88 degrees North latitude.
